# Data Sources as a Driver for Market-Oriented Tourism Organizations: a Bibliometric Perspective

**DOI:** 10.1007/s13132-023-01334-5

**Published:** 2023-05-25

**Authors:** Juan Vidal, Ramón A. Carrasco, Manuel J. Cobo, María F. Blasco

**Affiliations:** 1 Faculty of Commerce and Tourism, Complutense University of Madrid, 28003 Madrid, Spain; 2 Department of Management and Marketing, Faculty of Commerce and Tourism, Complutense University of Madrid, 28003 Madrid, Spain; 3 Department of Computer Science and Artificial Intelligence, Andalusian Research Institute in Data Science and Computational Intelligence (DaSCI) , University of Granada, Granada, 18071 Spain; 4 Department of Management and Marketing, Faculty of Commerce and Tourism, Complutense University of Madrid, 28003 Madrid, Spain

**Keywords:** Tourism, Data-driven, Market-oriented, Bibliometric analysis, Data sources, Data analysis

## Abstract

This paper presents a conceptual framework that accurately represents the current and future perspectives of data-driven companies in tourism by means of an analysis of the data sources used in the data-driven tourism research literature, as well as the research topics to which they are applied. For this purpose, a bibliometric analysis of data-driven tourism research is carried out. The framework of the study is all tourism-related publications whose research was based on data sources during the period 1982–2020. The results show some of the basic bibliometric performance indicators and the maps of science. The main themes of research interest are identified, and the conceptual evolution is obtained based on these maps. Three major thematic areas are identified: tourism research topics, information sources, and data analysis techniques. Based on these three thematic areas, the conceptual model of data architecture and processes of a data-driven organization in the tourism sector are obtained. An additional qualitative analysis of the three thematic areas is performed.

## Introduction

Digitalization has led many organizations in different sectors of activity to transform their operations by prioritizing data as a driver of their development. This change (Parra et al., 2022) has given rise to data-driven organizations (Davenport, 2015), organizations in which data becomes a key asset (Monino, 2021) in their business processes. Obviously, the aim is to use this data to have a better market orientation (Kohli & Jaworski, 1990). This orientation strategy must be based on the generation of market intelligence on current and future customer needs throughout the organization, must facilitate the dissemination of information across all departments, and must have the ability to convert this information into concrete market actions (Narver & Slater ,^1990^). There are generic conceptual architectures that identify the necessary pieces for this digital transformation necessary for the transformation of the business itself, in which aspects such as big data or artificial intelligence are perfectly integrated (Moreno et al., 2019).

The tourism sector has not been immune to this change and is a sector in which the use of data has transformed decision-making in tourism management. Tourism has been one of the sectors most affected by COVID-19, and this has accelerated this transformation. The raw material of this transformation is, obviously, data. The sources of information available in the tourism sector have grown significantly in the last decade. Through mobile devices, tourists are increasingly connected. Through their connections, they generate information, which we call “digital footprints”. This information, applied in the context of data protection, allows public and private organizations to make decisions for the intelligent development of tourist destinations, helping to obtain a 360° view both from tourists with the aim of anticipating their needs and adapting services to the visitor’s reality (Camilleri, 2020) and the competition, as market orientation requires. The data generated by mobile devices are only a part of the data generated by tourists (Li et al., 2018) since we also have the data generated in social networks, opinions, and comments on tourist services platforms, internet searches, online bookings, or card payments, among others. These data are the raw material of many analyses, studies, and researches (Bueno et al., 2021; Carrasco et al., 2017; Carrasco et al., 2014), without forgetting the data generated by organizations such as tourism statistics or open-data sources (Celdran-Bernabeu et al., 2018). There are, therefore, a wide range of data sources that are analyzed in a wide variety of studies.

This article is based on the assumption that any current market orientation of a tourism company, i.e., that aims to have an in-depth knowledge of both its customers and its competitors, must be based on data. Therefore, if we analyze the use of different data sources in the tourism sector, we will be able to draw a conceptual framework that accurately represents the current and future perspectives of data-driven companies in tourism. Therefore, the aim of this article is to obtain this conceptual framework through an in-depth view of the use of data sources in tourism research literature, as well as its temporal evolution and future perspectives. For this purpose, a bibliometric analysis of this type of research will be carried out. We will use the results of the bibliometric analysis to identify the main research themes of the scientific community and obtain insights and conclusions about the tourism research topics, the data analysis techniques used, and the sources of information employed. These insights will allow us to get a conceptual model of data architecture and processes of a data-driven organization in the tourism sector.

Bibliometrics is a useful tool for evaluating and analyzing the results of academic research. One of the main contributions of bibliometrics is the use of objective criteria to evaluate the research developed by researchers, and it is highly valued as a tool for measuring academic quality and productivity (Moed et al., 1995). Two main methods are commonly used to explore a given field of research: performance analysis and science maps (Noyons et al., 1999; Van-Raan, 2004). The objective of performance analysis is to assess the impact of the scientific output of different scientific actors. On the other hand, science maps, however, aim to show the conceptual, social, or intellectual structure of scientific research, as well as its evolution and dynamic aspects.

This article is structured as follows: In “Background of Data-Driven Architecture for Market-Oriented Organizations,” the background of generic data architecture for business is shown, i.e., the necessary components for data-driven business decision-making; in “Related Work,” the related work is presented. Thereafter, in the “Methodology,” the methodology used to achieve our objective is explained, and “Results” presents the results obtained after applying this methodology. Finally, some conclusions and future work are drawn in “Conclusions and Future Work.”

## Background of Data-Driven Architecture for Market-Oriented Organizations

In the previous section, we introduced the importance of a correct market orientation. We can identify three main dimensions of a market-oriented strategy (Kohli & Jaworski, 1990): generation of market intelligence, which consists of the responsibility of the entire organization to obtain a marketing information system on the present and future needs of customers, as well as those of distributors, suppliers, pressure groups, competitors, and the macroenvironment in general. Dissemination of information, among the different areas of the organization, so that they work together towards the same objective. Response to the information, so that this knowledge obtained and disseminated is converted into actions that allow obtaining competitive advantages in the organization.

In a market-oriented business strategy, data plays a fundamental role and the way it is stored, managed, and analyzed is crucial. It is necessary to have the right data architecture and processes in place to effectively implement these strategies. This architecture should have all the necessary components for data-driven business decision-making.

**Fig. 1:**
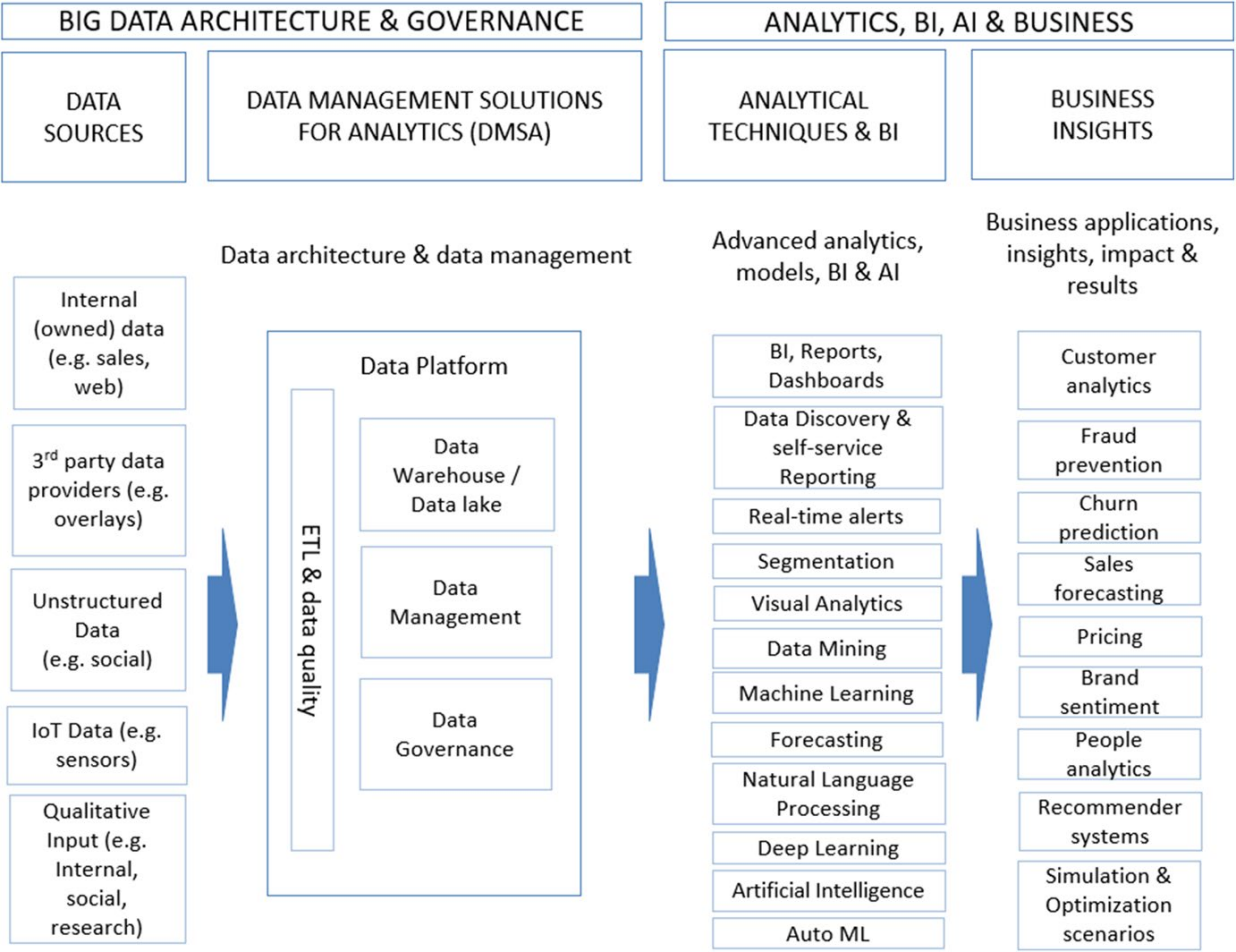
Conceptual model of data architecture and processes of a data-driven organization based on Moreno et al. (2019)

Figure 1 shows a conceptual model of the data and process architecture (Moreno et al., 2019) that a data-oriented organization should have. This model considers several differentiated areas: firstly, the data sources are represented; secondly, the area of data management and data warehousing; thirdly, the area of data analytics, business intelligence, and artificial intelligence; and finally, the applications and business insights generated.

The different components of the conceptual model are explained in more detail below:

- Data sources: It would include the internal data of the organization such as the contents in the operative processes of enterprise systems, those related to bidirectional communications with customers and society in general commonly supported in collaborative and social CRM, third-party data, and qualitative data including those from typical secondary sources.
- Data management solutions for analytics: They are the databases specifically designed for analysis, and they would include much of the raw data discussed, through the so-called ETL (extraction, transformation, and loading) processes. The raw data is stored in the data lake and the processed, cleaned, and enriched data is stored in the data warehouse.
- Analytical techniques and BI: This component contain business intelligence and advanced analytics solutions. The advanced analytics is supported by data mining or data science of which modeling phase is, in turn, supported largely by artificial intelligence, and more specifically, by machine learning systems.
- Business insights: This component is related to business decisions based on the knowledge discovered in the previous component. This knowledge will be applied to improve the relationship with the customer, it will materialize in data-driven business decisions.

As mentioned above, this work aims to instantiate this generic conceptual architecture in the tourism sector landscape drawing what parts are actually being used in the sector in each of its layers.

## Related Work

Some authors have conducted a review of bibliometric studies in the field of tourism (Koseoglu et al., 2016) which concludes that there are many more systematic literature review studies than bibliometric studies, also pointing out that the number of analyses with relational bibliometric techniques is relatively low.

Considering the available literature on bibliometric studies, we find that several authors have conducted bibliometric studies in thematic areas related to tourism from different points of view.

First, we find authors who have conducted general studies on tourism research from different perspectives, some authors have focused on analyzing the quality of journals (Hall ,^2011^), other authors focus on analyzing the contribution of authors and the relationships between authors based on citations (Benckendorff & Zehrer, 2013), and other authors (Mulet-Forteza et al., 2019) add to the previous perspectives an analysis of the main research topics.

Secondly, we find authors who have conducted studies on specific thematic areas of relevance in the tourism sector such as tourism innovation (Omerzel ,^2016^), sustainable tourism (Ruhanen, 2015; Martinez-Martinez et al., 2022), tourism promotion (Florido-Benitez, 2022), medical tourism (de la Hoz-Correa, et al., 2018), economic impact (Comerio & Strozzi, 2019), rural tourism (Ruiz-Real et al., 2020), adventure tourism (Cheng et al., 2018), or gastronomic tourism (Okumus, 2018), among others.

Third, we focus on bibliometric studies that focus on data-driven research. That is, research in the field of tourism with an approach based on data and analytical techniques. It is on this type of studies that the article we propose will focus on. It should be noted that there are also literature reviews (Li et al., 2018; Mariani et al., 2018; Rahmadian et al., 2022; Samara et al., 2020; Li et al., 2022; Nyanga et al., 2020; Mariani & Baggio, 2022) in this field. In this line, we find bibliometrics focused on the use of artificial intelligence in tourism (Kirtil & Askun, 2021; Sharma et al., 2022). This bibliometric uses the Scopus database and identifies the most productive authors and institutions, as well as the most relevant research topics. The evolution of publications over the periods analyzed is shown. We propose in our article a different perspective covering all tourism research based on data sources and not only those using artificial intelligence techniques, analyzing both techniques, data sources, and research topics, using Web of Science (WoS) as a database. There are also authors (Lv et al., 2021) who analyze the use of artificial intelligence by adding the use of big data. This study shows the temporal evolution of publications, main keywords, and distribution of publications by journals. It also identifies and classifies the main data sources and the main research topics. Likewise, some authors (Gómez & Gil, 2020) carried out a bibliometric analysis about knowledge management and value creation through big data in the tourism sector. Similar to what was explained in the previous article, we propose in our article a different perspective covering all research in tourism based on data sources and not only those using artificial intelligence techniques. Regarding bibliometric analysis, we include techniques such as science maps and conceptual evolution based on them, which are techniques not addressed in the aforementioned article. On the other hand, there are also bibliometric studies that exclusively analyze a specific type of data source, such as studies focused on data from social networks (Nusair et al., 2019), on user-generated data in online platforms (Muritala et al., 2020; Mukhopadhyay et al., 2022; Akbari et al., 2022), or on data generated by mobile devices (Chantre-Astaiza, 2019). Studies of this type exclusively analyze one type of data source; in the article we propose, we analyze the different types of data sources used in data-driven tourism research.

Table 1 shows a summary of the articles discussed in the third paragraph. That is, literature reviews or bibliometric studies that focus on data-driven research.

**Table 1:** Summary of literature reviews or bibliometric studies that focus on data-driven research

Article reference	Objective	Type of review
Li et al., 2018	Big data in tourism	Literature review
Mariani et al., 2018	Business intelligence and big data in hospitality and tourism	Systematic literature review
Rahmadian et al., 2022	The use of big data for sustainable tourism	Systematic literature review
Samara et al., 2020	Artificial intelligence and big data in tourism	Systematic literature review
Li et al., 2022	Big data in China tourism research	Systematic literature review
Nyanga et al., 2020	Enhancing competitiveness in the tourism industry through the use of business intelligence	Literature review
Mariani and Baggio, 2022	Big data and analytics in hospitality and tourism	Systematic literature review
Kirtil and Askun, 2021	Artificial intelligence in tourism	Bibliometric
Sharma et al., 2022	Artificial intelligence in tourism	Bibliometric
Lv et al., 2021	Big data and artificial intelligence literature in hospitality and tourism	Bibliometric
Gómez and Gil., 2020	Knowledge management and value creation through big data in the tourism sector.	Bibliometric
Nusair et al., 2019	Social media in hospitality and tourism research	Bibliometric
Muritala et al., 2020	Online review research in tourism and hospitality	Bibliometric
Mukhopadhyay et al., 2022;	Electronic word of mouth (e-WOM) research	Bibliometric
Akbari et al., 2022	The evolution of e-WOM from the past to the future	Bibliometric
Chantre-Astaiza et al., 2019	Tourist mobility. Mobile device data	Bibliometric
Our proposal	Data sources used in data-driven tourism research	Bibliometric

After reviewing the related articles, we have not found any previous work that has attempted to provide an overview of the current landscape of data-driven companies in tourism by defining a conceptual framework for these data-driven companies. Besides, the proposed article differs from the more related, already mentioned, for several reasons: broad and updated time frame (1982–2021) of WoS articles. It also differs in the analysis of the research themes and their conceptual evolution over the period, all using the science mapping tools provided by SciMAT (Cobo et al., 2012), which allows a more in-depth description of the themes. A broad approach to tourism research based on data sources is proposed, analyzing not only the research themes, but also the data sources used and the data analysis techniques used.

## Methodology

The methodology used in this paper is inspired on Cobo et al. (2011), Carrasco-Aguilar et al. (2022), Galán et al. (2022), and Minhas and Sindakis (2021).

**Fig. 2:**
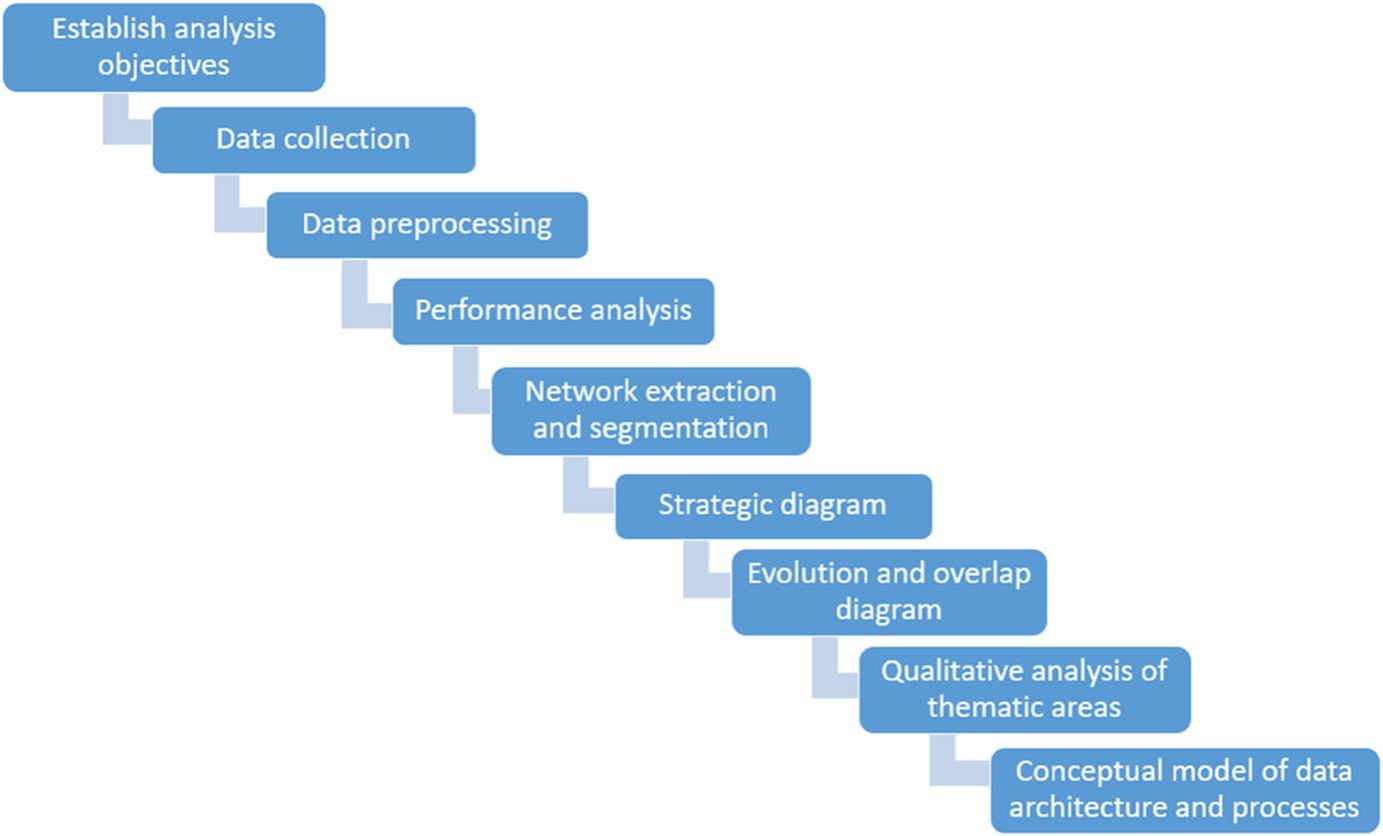
Main steps of the methodology

Figure 2 shows schematically the steps to be performed.

### Establish Analysis Objectives

The objective of this study is to get a conceptual model of data architecture and processes of a data-driven organization in the tourism sector based in a bibliometric analysis. We will use the results of the bibliometric analysis to identify the main research themes of the scientific community and obtain insights about the tourism research topics, the data analysis techniques used, and the sources of information employed. These insights will allow us to get this conceptual model of data architecture and processes. In summary, we have a primary objective, which is to obtain the conceptual model of data architecture and processes, and a secondary objective, which is the bibliometric analysis.

### Data Collection

The scientific papers published in the field of study must be collected. The documents were downloaded from WoS. For this purpose, the advanced search was used with the following search equation:

TS = ((“tourism” OR “tourist*” OR “hospitatility” OR “hotel*”) NEAR/3 (“data source*” OR “information source*” OR “dataset*” OR “information system*” OR “database*” OR “data warehouse*” OR “data lake*”))

Obtaining a total of 794 documents in that time frame (1982–2021). The data were downloaded on 21/09/2022. In this research, to perform the bibliometric analysis, we have used the SciMAT software (Science Mapping Analysis Software Tool) (Cobo et al., 2012) since it integrates everything we require for this research and because of its free and open-source nature.

### Data Pre-Processing

The raw documents were downloaded from WoS as plain text including the complete record. This document in txt format was entered into SciMAT to build the database from which the bibliometric analysis will be performed.

To increase the quality of the data, a refinement was performed. Keywords have been normalized by merging them in their plural and singular forms, words have also been merged with their corresponding synonyms, and several keywords that refer to the same concept have been identified by using the Levenshtein distance in SciMAT.

Then, using SciMAT’s period manager, two consecutive periods of time were established to show the conceptual evolution in the analysis of science maps. By performing a previous analysis of the distribution of publications over time, it was decided to divide the original time frame (1982–2021) into two periods ensuring that both periods had a similar number of documents. The data were therefore divided into two consecutive time periods: 1982–2015 with 393 papers and 2016–2021 with 401 papers.

### Bibliometric Performance Analysis

In this phase, the bibliometric analysis of performance was performed, in which publications are analyzed from multiple perspectives: evolution over time of the number of publications, journals with the highest number of publications, geographical distribution of publications, and most productive authors. Graphs obtained in this bibliometric analysis are made with Python programming language and Matplotlib package using SciMAT’s data.

### Network Extraction and Segmentation

Next, science maps were made, which are spatial representations of the relationships that exist between documents, authors, fields, or disciplines. They have been widely used in the scientific literature in very diverse fields to show these relationships graphically (Huang & Chang, 2014; Liu et al., 2012).

We applied a co-word analysis (Callon et al., 1983) based on the keywords of the documents for each period. We then performed keyword clustering using the simple center algorithm (Coulter et al., 1998), which locates networks of keywords that are closely linked to each other and correspond to centers of interest or research problems of significant interest among researchers. This similarity between keywords is evaluated using the equivalence index (Coulter et al., 1998): *eij* = *cij*^2^/*cicj* where *cij* is the number of documents in which two keywords *i* and *j* coexist and *ci* and *cj* represent the number of documents in which each appears.

### Strategic Diagram

In this phase, through the strategic diagram (He, 1999) and the thematic network, the most prominent themes are visualized. Each of these themes is characterized by two measures (Coulter et al., 1998): centrality and density. Centrality measures the degree of interaction of a network with other networks, defined as =10 ×  ∑ *ekh* , where *k* is a keyword belonging to that same theme and *h* is a keyword belonging to other themes.

**Fig. 3:**
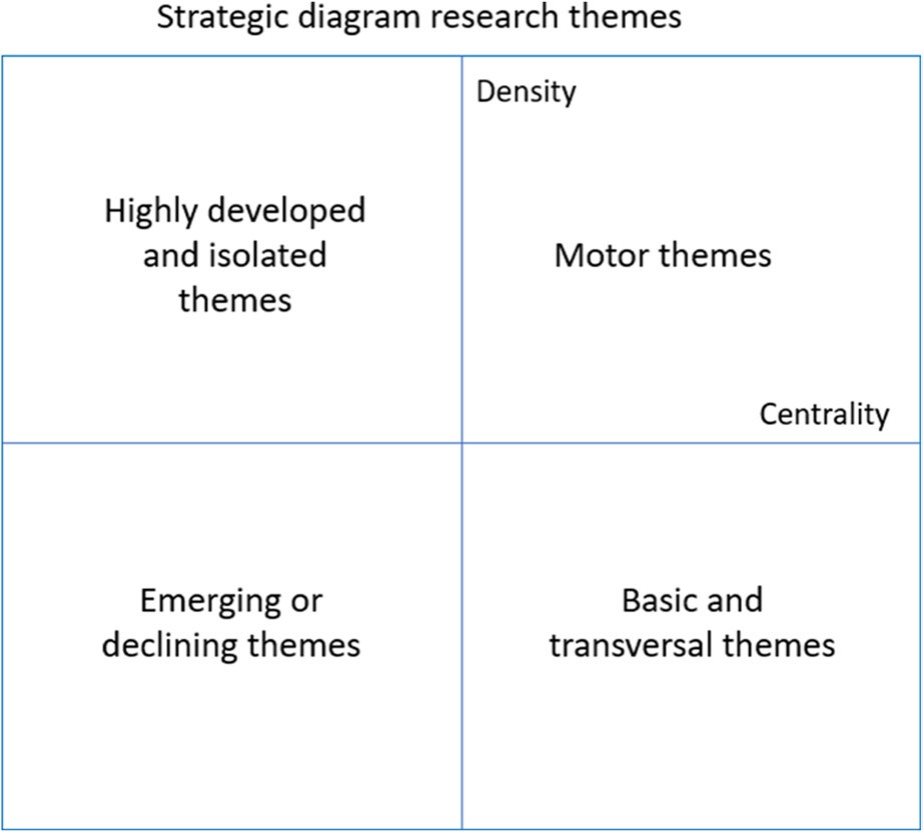
Strategic diagram (Cobo et al., 2012)

On the other hand, density measures the internal intensity of the network and can be defined as *d* = 100 × (  ∑ *eij*/*w*  ), where *i* and *j* are keywords belonging to the theme, and *w* is the number of keywords in the theme. With these two measures, we can visualize the research themes through a two-dimensional strategic diagram (Fig. 3) where the research themes are classified into four groups.

- In the upper right quadrant are the themes that have a strong centrality and high density; they are, therefore, the driving themes of our research field. They are well-developed and important for the structure of the research field.
- In the upper left quadrant are located the highly specialized but peripheral themes. These are themes that are of marginal importance to the field, as these themes are internally well developed but their links to other external themes are very weak.
- In the lower left quadrant represented the emerging or disappearing themes, since they have a low density and centrality. They are underdeveloped and marginal themes.
- In the lower right quadrant, transversal and general themes are represented, i.e., basic, relevant to the field of research, but not very developed.

The performance analysis is performed as a complement to the scientific mapping work, for which the following bibliometric indicators are used: number of published papers, number of citations, and h-index (Alonso et al., 2009; Hirsch, 2005; Martínez et al., 2014). To evaluate these performance indicators, the SciMAT program, using a function, assigns a set of documents to each detected theme; specifically, SciMAT returns the algebraic union of the set of documents associated with the keywords of the theme. A document has several keywords, since each of them can be associated with a different theme, so that a document could be associated with several themes.

Once the strategic diagram has been obtained, it is interesting to identify the main thematic areas into which we can classify the themes obtained. This classification into thematic areas will allow us to better understand the bibliometric results.

### Evolution and Overlap Diagram

In this phase, research themes are detected over a period of time, after which the general areas of evolution, their origins, and interrelationships are analyzed. For this purpose, the inclusion index (Sternitzke & Bergmann, 2009) is used, which detects conceptual links between research themes from different periods. Subsequently, a bibliometric map is made with the thematic evolution in two time periods; the themes can be unlinked, linked by continuous lines or dotted lines. Furthermore, depending on whether the value of the inclusion index is higher or lower, so will be the thickness of the lines linking the linked themes (Cobo et al., 2015).

### Qualitative Analysis of Thematic Areas

In addition to the performance measures and evolution maps obtained, it is worth carrying out a qualitative analysis of the thematic areas identified based on the main keywords of these areas.

### Conceptual Model of Data Architecture and Processes of a Data-Driven Organization in the Tourism Sector

Once the bibliometric analysis has been carried out and the main thematic areas have been identified and analyzed, we will use these insights to get the conceptual model of data architecture and processes of a data-driven company in the tourism sector, which is our priority objective as mentioned above.

## Results

In this section, we obtain some of the basic bibliometric performance indicators and the maps of science. The main themes of research interest are identified and the conceptual evolution is obtained based on these maps. Three major thematic areas are identified and based on these thematic areas the conceptual model of data architecture and processes of a data-driven organization in the tourism sector is obtained. An additional qualitative analysis of the three thematic areas is performed.

### Bibliometric Performance Analysis

**Fig. 4:**
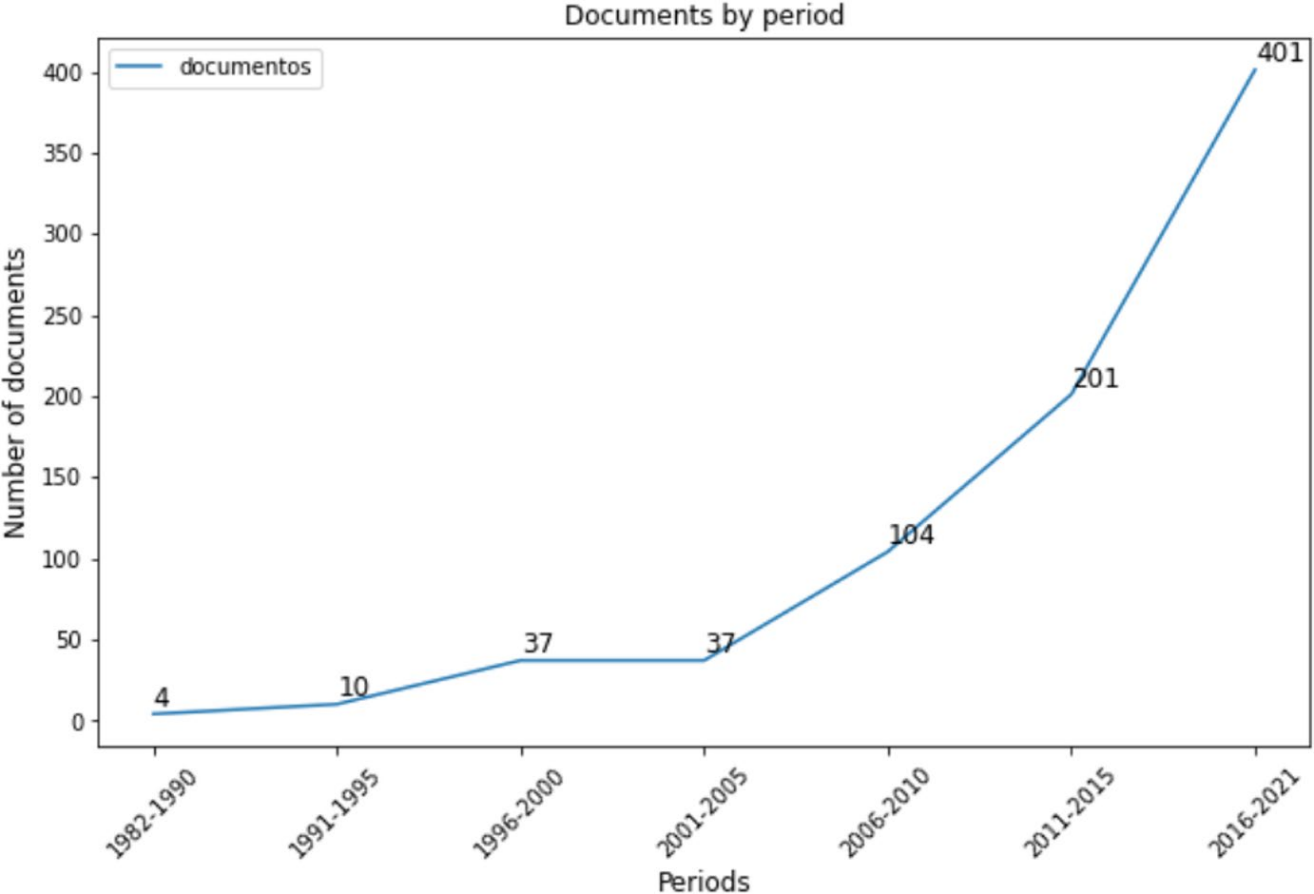
Time series representing number of documents by period

First, we reviewed the temporal evolution of the publications. The distribution of documents by year is shown in Fig. 4. The table shows seven 5-year periods, except for the initial period which covers 8 years due to the low number of documents in those years and the last period with 6 years to go until 2021. As can be observed, the number of publications has been increasing over the periods. The first two periods have a low number of documents, but there is already an increase between the first and second period. The number of documents remains stable in the third and fourth periods and rises again considerably in the last three periods. We can see that between the fifth and seventh periods, the number of documents quadrupled.

**Fig. 5:**
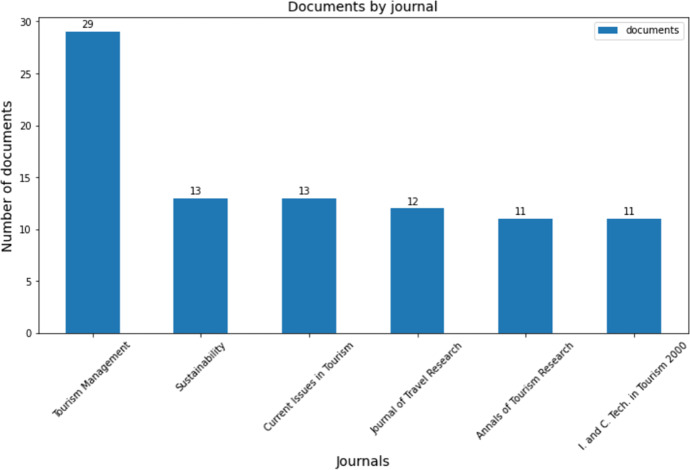
Bar chart containing journals with more than 10 publications

If we focus on the distribution of publications by journals, Fig. 5 shows a bar chart with journals with more than 10 publications. The journal “*Tourism Management*” stands out with 29 publications.

**Fig. 6:**
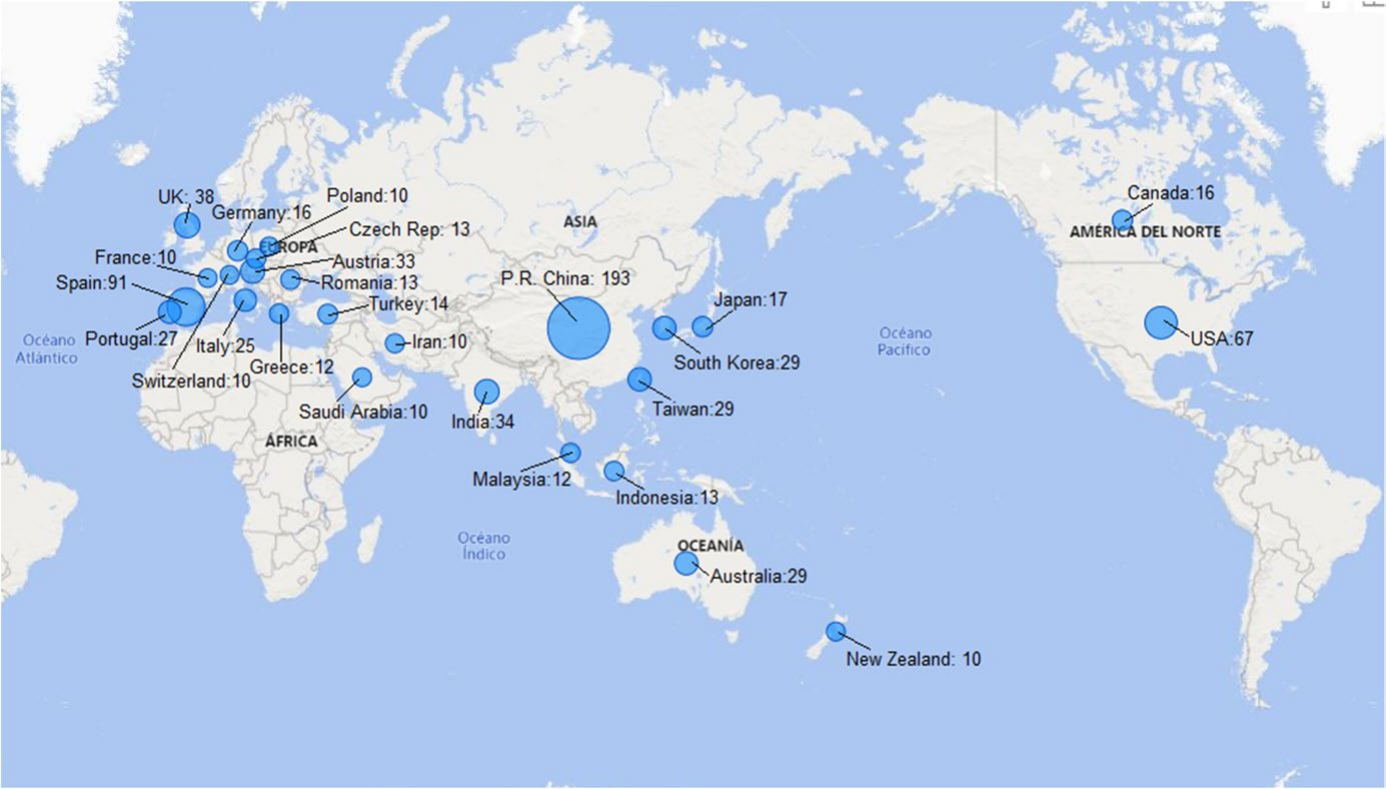
World map with countries with more than 10 publications

Regarding the distribution of documents by country, Fig. 6 shows a world map with countries with more than 10 publications. China is the leader in the number of documents published, followed by Spain and the USA.

With regard to the authors, we find that the distribution is very evenly distributed. It is observed that there is no author with a very high number of publications. As for the most productive authors, Pröll, Birgit; followed by Law, Rob, and Retschitzegger, Werner. Pröll and Retschitzegger have joint publications at the Johannes Kepler University of Linz, while Law belongs to the University of Macau.

### Strategic Diagram

In this section, we get the spatial representations of the relationships that exist between documents following the methodology of Cobo et al. (2011) based on a co-word analysis as described in the previous section. First, we obtain the content analysis of published papers for each of the two periods; second, the evolution map between periods; and finally, we make a qualitative analysis of the results.

The analysis is based on two periods (1982–2015 and 2016–2020), and the descriptions will be supported by tables and strategic diagrams. In the strategic diagrams, the size of the sphere of each research theme is proportional to the number of papers published on that theme.

## First Period (1982–2015)

**Fig. 7:**
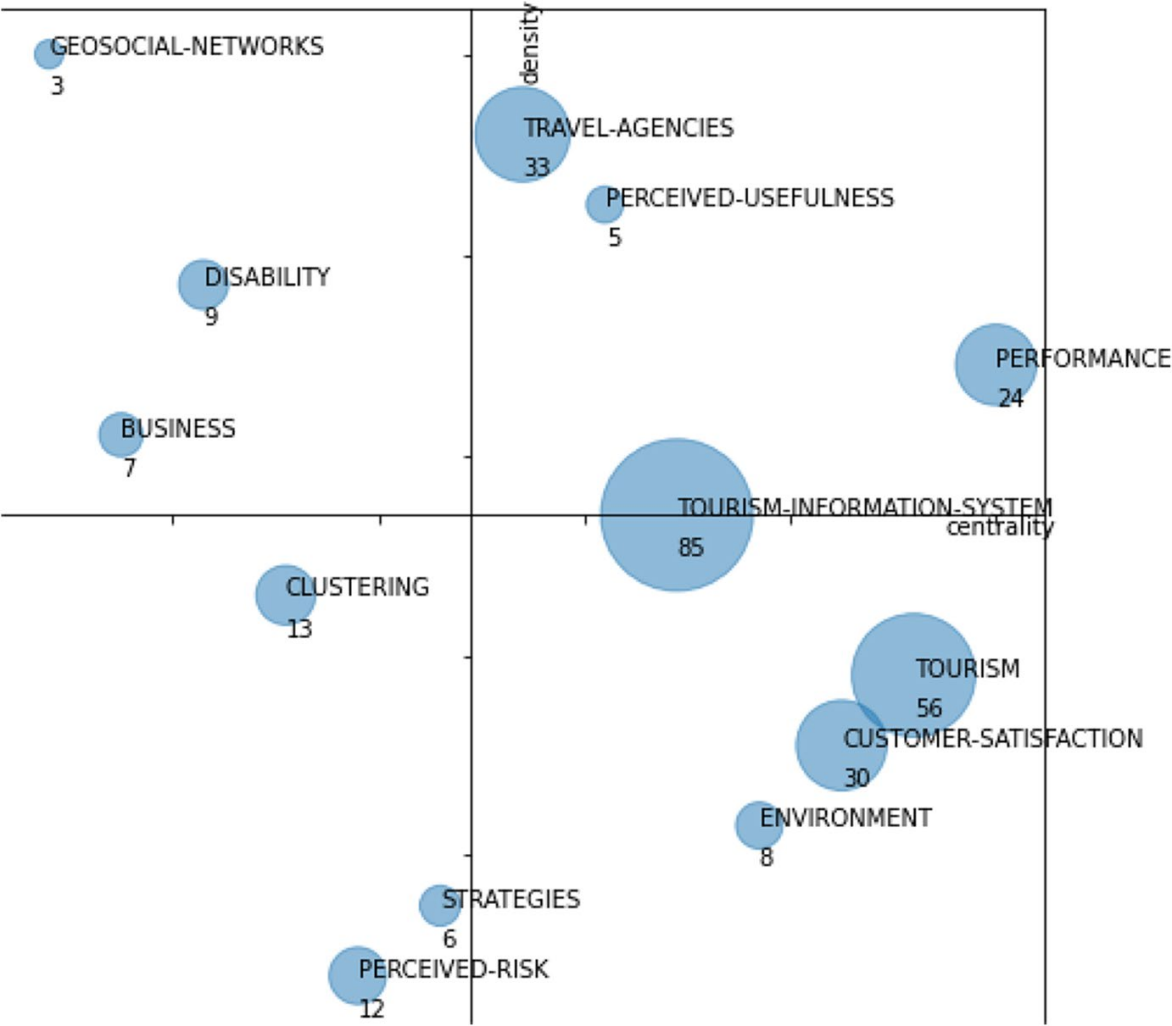
Strategic diagram for the period 1982–2015. The number of documents in each theme is shown

As shown in Fig. 7, this first period contains 13 research themes: “performance,” “customer-satisfaction,” “tourism-information-system,” “tourism,” “travel-agencies,” “geosocial-networks,” “perceived-usefulness,” “disability,” “business,” “clustering,” “strategies,” “environment,” and “perceived-risk.”

Table 2 shows the performance indicators: number of documents, citations obtained by these documents, and the h-index. According to those indicators, we could identify as main themes the following: “tourism-information-system,” “tourism,” “travel-agencies,” “customer-satisfaction,” and “performance.” Between these 5 themes, they account for 74% of the total number of citations and 78% of the documents. The h-index of these themes is also higher, although there is not as notable a difference as in the number of citations or documents.

**Table 2:** Performance indicators by themes in the period 1982–2015

Themes	Number of documents	H-index	Citations
Tourism-information-system	85	10	449
Tourism	56	16	891
Travel-agencies	33	11	904
Customer-satisfaction	30	19	1295
Performance	24	16	1012
Clustering	13	6	215
Perceived-risk	12	8	266
Disability	9	6	98
Environment	8	5	142
Business	7	4	263
Strategies	6	6	199
Perceived-usefulness	5	5	362
Geosocial-networks	3	1	28

These main themes are described below:

The “tourism-information-systems” theme is a driving theme; it is the theme with the largest number of documents, although it has less weight in terms of citations and h-index. It focuses on the sources of information used in tourism, with emphasis on sources of geographic information data (GIS), and to a lesser extent and with much less weight than the previous one, on statistical information systems. It also includes various issues that refer to technological issues related to information sources such as the semantic web, ontologies, and integration processes.

Another outstanding driving theme is “performance”; this is the second theme with the highest number of citations. It focuses on issues related to measuring competitiveness and impact from different perspectives of the tourism industry, highlighting themes such as service quality and to a lesser extent productivity or strategies. The competitiveness and service quality of tourism destinations are one of the main objects of study in data-driven analyses in the tourism sector.

The third leading driving theme is “travel-agencies”; it is the third theme with the highest number of citations. It includes themes related to travel agencies, the Internet, or the use of images of tourist destinations, reflecting the growing importance of the Internet in tourism, as well as the use of images of destinations. It also includes issues related to relational databases, reflecting their use for the storage and analysis of structured information in the tourism sector.

The theme “customer-satisfaction” is a basic and transversal theme. It is the theme with the highest number of citations and the second-highest h-index. It covers a wide range of issues, but those related to tourist behavior, such as loyalty or motivations, stand out. Similarly, and related to the previous ones, there are issues related to the search for information. Tourist behavior and satisfaction is another of the main topic of analysis in data-driven tourism research.

The “tourism” theme is a basic and transversal theme. It is the second theme with the highest h-index, equal in this position with performance. As is usual for the themes in this quadrant, it is a fairly general theme. It includes wide-ranging issues related to management or services, such as market segmentation or distribution channels.

Looking at all the themes identified in this period, it makes sense to group them into three broad thematic areas, namely:

- Tourism research topics: It covers this research topics from a business perspective. The themes “customer-satisfaction,” “tourism,” and “performance” focus on topics under study in data-driven tourism research and is included in this thematic area. Other themes of the period, with a weight in number of documents, h-index, and number of citations much lower than the previous ones, we find a similar classification: “perceived-risk,” “disability,” “environment,” “business,” “strategies,” and “perceived-usefulness.”
- Information sources: It includes themes related to information system sources. The theme “travel-agencies” and “tourism-information-system” belongs to this area. The theme “geosocial-networks” also belongs to “information sources.” We further include those related to geolocated social networks.
- Data analysis techniques: This area covers themes related to statistical techniques and data science that are applied in research. The “clustering” topic belongs to this area.

**Fig. 8:**
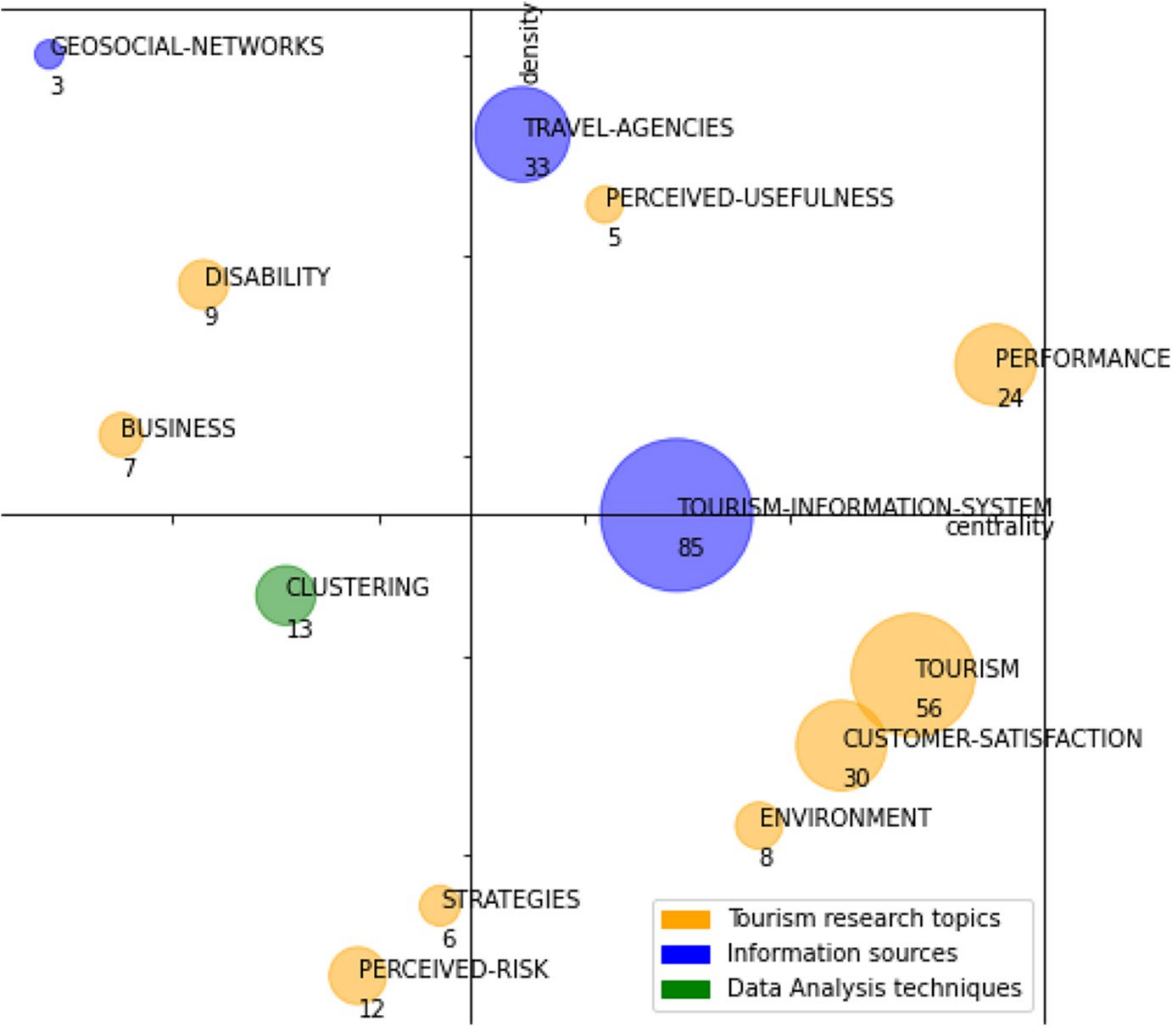
Strategic diagram for the period 1982–2015 classified by thematic area. The number of documents in each theme is shown

Figure 8 highlights in different colors the themes of each of the thematic areas mentioned above, and Table 3 shows the performance indicators based on these three thematic areas.

**Table 3:** Performance indicators by thematic area in the period 1982–2015

Thematic areas	Number of documents	Citations
Tourism research topics	157	4528
Information sources	121	1381
Data analysis techniques	13	215

## Second Period (2016–2021)

**Fig. 9:**
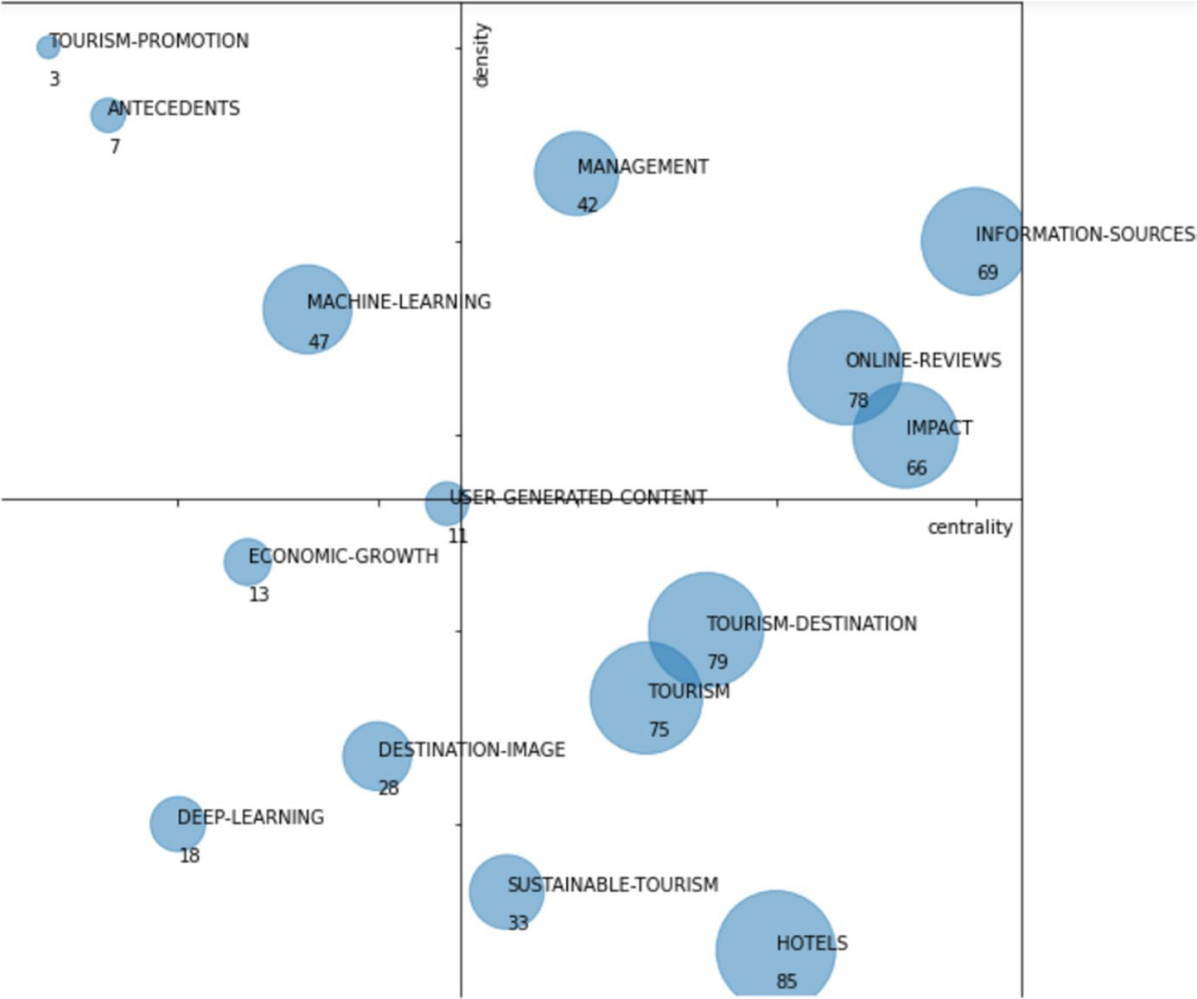
Strategic diagram for the 2016–2021 period. The number of documents in each theme is shown

As can be observed in Fig. 9, this first period contains 15 research themes: “antecedents,” “deep-learning,” “destination-image,” “economic-growth,” “hotels,” “impact,” “information-sources,” “machine-learning,” “management,” “online-reviews,” “sustainable-tourism,” “tourism,” “tourism-destination,” “tourism-promotion,” and “user-generated-content.”

Table 4 shows the performance indicators: number of documents, citations obtained by these documents, and the h-index. According to those indicators, we could highlight as main themes the following: “hotels,” “tourism-destination,” “online-reviews,” “tourism,” “information-sources,” and “impact.” These 6 themes together account for 73% of the total number of citations and 69% of the documents. The h-index of these topics is also higher, although the difference is not as great as in the number of citations or documents. The main themes are described below.

**Table 4:** Performance indicators by themes in the 2016–2021 period

Themes	Number of documents	H-index	Citations
Hotels	85	10	513
Tourism-destination	79	14	1072
Online-reviews	78	15	979
Tourism	75	12	597
Information-sources	69	14	841
Impact	66	13	655
Machine-learning	47	7	257
Management	42	9	364
Sustainable-tourism	33	7	175
Destination-image	28	8	219
Deep-learning	18	7	236
Economic-growth	13	5	173
User-generated-content	11	6	162
Antecedents	7	5	86
Tourism-promotion	3	2	6

These main themes are described below:

The theme “online-review” is a driving theme; it is the third theme with the highest number of documents and the second in terms of citations. It focuses on information generated by the tourist, either through ratings or opinions on intermediation platforms in the contracting of tourism services, satisfaction surveys, comments on social networks, exchange of information in virtual communities, or e-mails. Data of this type are more generally classified in UGC (user-generated content) data (Li et al., 2018). Related to the above appear issues related to data analytics. It reflects the great relevance that the analysis of tourist-generated information has acquired for tourism, both because technology makes it possible to capture and store it and because of the shift towards a more focused approach to analyzing tourist behavior and experience.

The “information-sources” theme is a driving theme; it is the third theme with the highest number of citations and the second in terms of h-index equals with “tourism-destination.” It contains issues related to the different data sources differentiated by type and origin. It covers data from travel, Internet search engines, images, tourist behavior, or hotel reservations. It reflects the wide range of available data sources that has been growing in variety over the last decade.

The “impact” theme is a driving theme; it contains differentiated issues, including the impact of social networks on tourism, which are becoming increasingly relevant, and on the other hand, a classic topic such as the impact of tourism on the economy, with emphasis on the economy of the countries. On the other hand, it contains issues related to the hospitality industry, distribution channels, and online tourist agencies.

The theme “tourism-destinations” is a basic and transversal theme; it is the theme with the highest number of citations, the second-highest h-index equals with “information-sources” and the second highest number of documents. It focuses on analyzing tourism destinations from the perspective of demand, tourist behavior, and experience. Current issues such as smart tourism, which is based on technology to improve services and the tourist experience, appear. The analysis of tourism destinations from different perspectives is one of the central themes in data-driven tourism research.

The theme “hotels” is a basic and transversal theme; it is the theme with the highest number of documents. It contains issues related to service quality, revenue management, services, industry, performance, and productivity. It is also related to the airbnb issue reflecting current changes in accommodation booking.

The theme “tourism” is a basic and transversal theme. As it is a very general topic, it is related to a wide range of issues, like determinants, gender-differences, price, social networks, and segmentation.

As a summary and using the thematic areas used in the first period, we see that the themes: “tourism-promotion,” “antecedents,” “tourism-destination,” “impact,” “management,” “tourism,” “economic-growth,” “sustainable-tourism,” and “hotels” belongs to “tourism research topics” thematic area. The themes “information-sources,” “online-reviews,” “user-generated-content,” and “destination-image” belongs to “information sources” thematic area and finally “machine-learning” and “deep-learning” belongs to “data analysis techniques.”

**Fig. 10:**
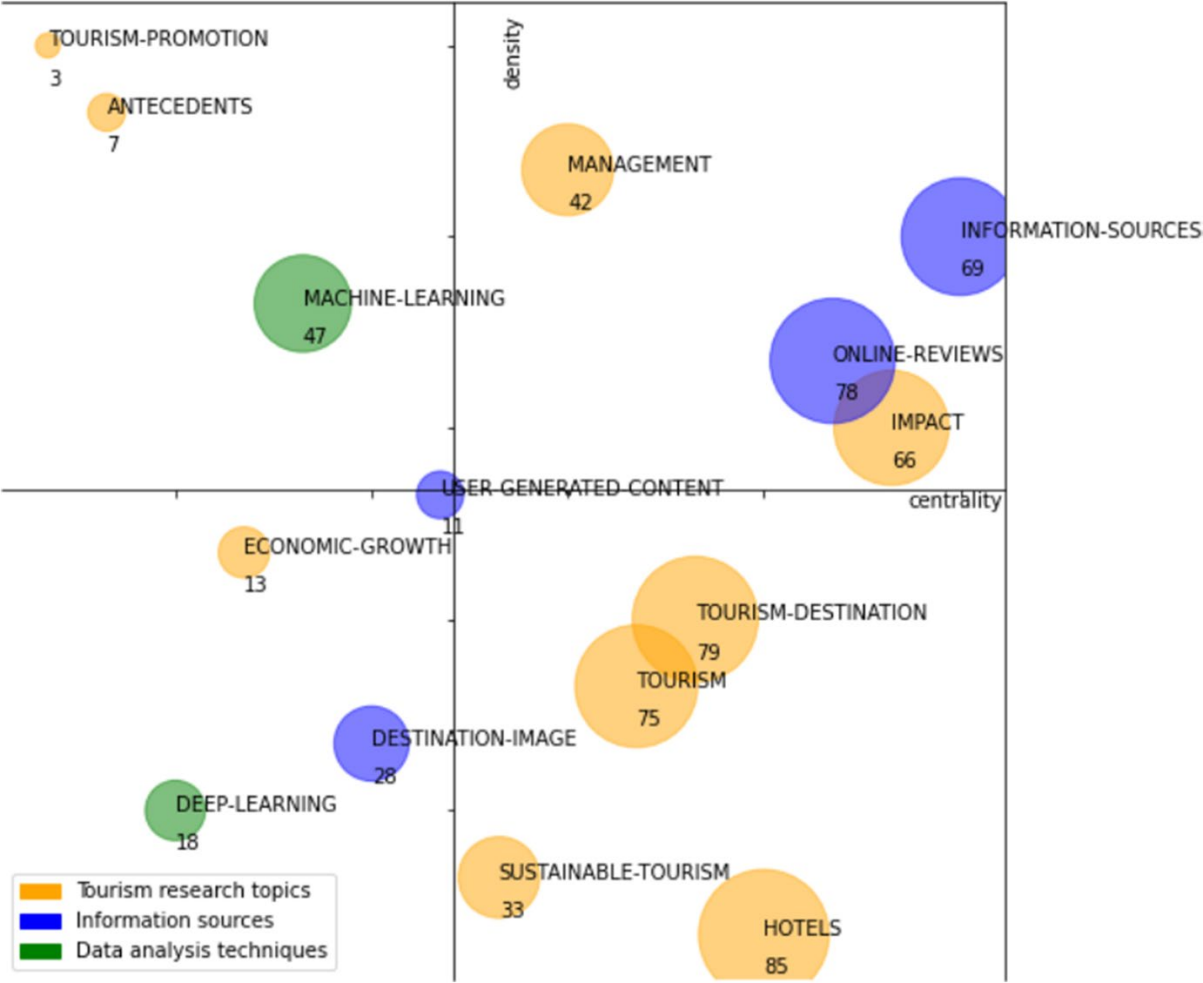
Strategic diagram for the period 2016–2021 classified by thematic areas. The number of documents in each theme is shown

In Fig. 10, the themes in each of the thematic areas are highlighted in different colors, and Table 5 shows the performance indicators based on these three thematic areas.

**Table 5:** Performance indicators by thematic areas in the period 2016–2021

Thematic areas	Number of documents	Citations
Tourism research topics	403	3641
Information sources	186	2201
Data analysis techniques	65	493

### Evolution and Overlap Diagram

An analysis of the themes detected in each time period has been developed taking into account the keywords and their evolution over time; for this purpose, SciMAT has been used again.

## Overlapping map of SciMAT shows that 12% of keywords are shared between periods.

**Fig. 11:**
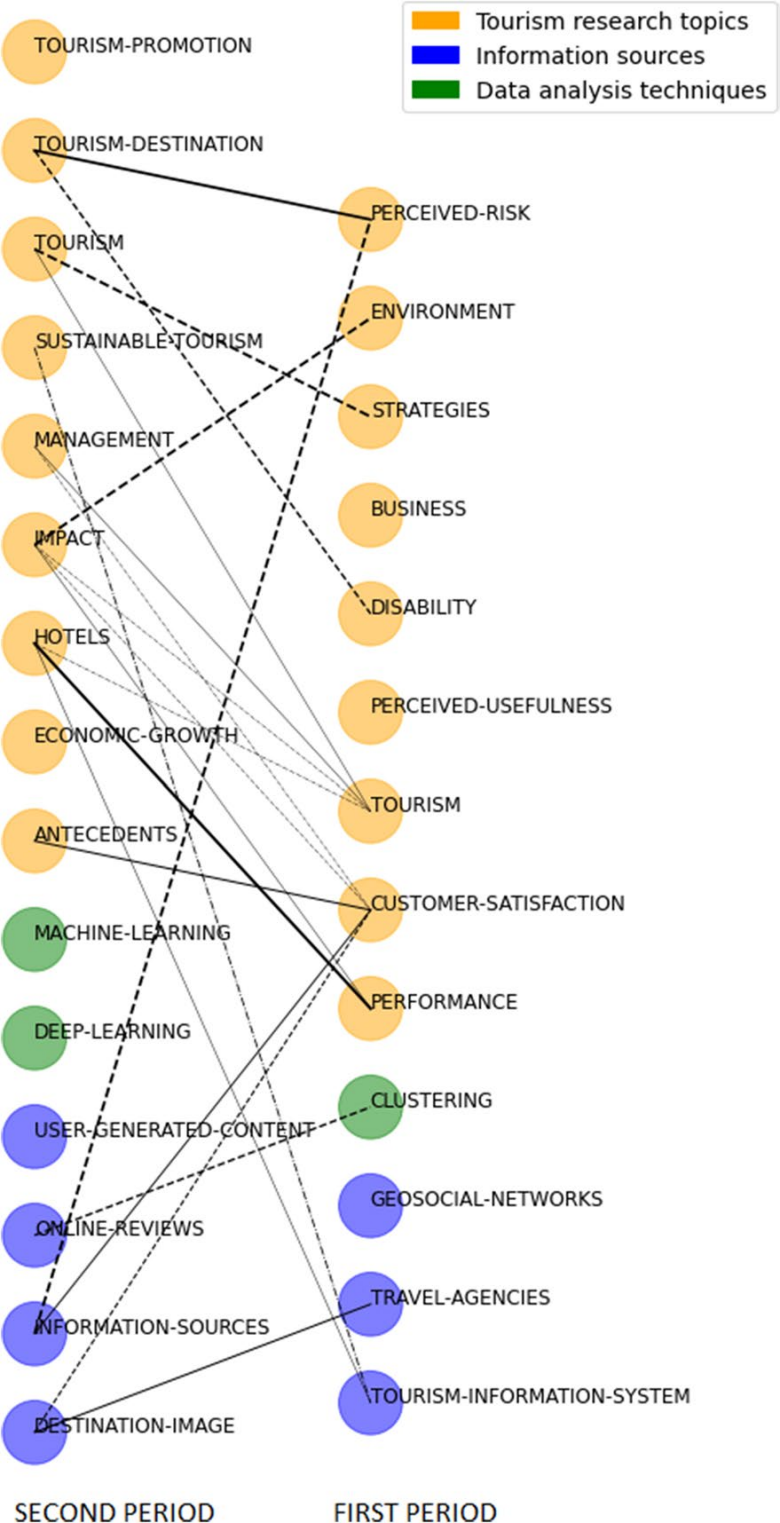
Conceptual evolution maps between periods

The conceptual evolution is shown in Fig. 11. In this map, continuous lines mean a thematic nexus, a dotted dashed line means that the linked themes share keywords different from the name of the themes, the thickness of the line is proportional to the inclusion index, the size of the sphere is proportional to the number of documents hosted by each theme, and the color shows the thematic area to which the themes belong.

In the evolution map, it is interesting to review the evolution of the thematic areas. Most of the themes of the thematic area “tourism research topics” connect with themes of the same area. The clear difference in themes between the first, and the second period reflects the change in research topics that has taken place between the two periods. The theme “impact” which belongs to the second period is an outstanding theme as it connects with four themes of the first period, of which, the strongest link is with “environment,” all of them from the thematic area “tourism research topics”. Also noteworthy in the thematic area of “tourism research topics” is the strong link between topics such as “perceived-risk” from the first period with “tourism-destination” from the second period, and the link between “strategies” from the first period and “tourism” from the second period the link between “performance” from the first period and “hotels” from the second period.

Regarding the thematic area of “data analysis techniques,” we see the themes “machine learning” and “deep learning” in the second period appears as a new themes with no link to the previous period, reflecting the disruptive emergence of these types of advanced data analytics techniques.

Finally, the evolution of the theme in the “information sources” thematic area reflects the change in the type of information sources used between the first and second periods. In the second period, new sources of information appeared, such as “online-reviews,” “destination-image,” and “user-generated-contents.” These new sources of information are generated by tourists themselves on different digital platforms and it is a type of information whose use has grown substantially.

### Qualitative Analysis of Thematic Areas

In addition to the performance measures and evolution maps obtained, it is worth highlighting some specific aspects that we detail based on the three thematic areas mentioned above. For this purpose, we deepen the analysis by reviewing the keywords contained in the different themes as a result of the co-word analysis. Also, we integrated each thematic area into the conceptual model of Fig. 1.

#### Tourism Research Topics

Keywords related to this thematic area include: “impact,” “tourism-destination,” “customer-satisfaction,” “customer-behaviour,” “travel,” “hospitality,” “management,” “hotels,” “word-of-mouth,” “performance,” and “sustainable-tourism.” Some of them are quite generic and associated with the tourism sector, such as “travel,” “hospitality,” or “hotels.”. On the other hand, others are more focused on specific topics such as “customer-satisfaction” and “customer-behaviour,” which reflect the growing interest in analyzing tourist satisfaction and behavior. There is a notable increase in research in this category between the first and second period. Another group of keywords can be “management,” “impact,” or “performance,” keywords commons in studies oriented to measure economic performance and from other points of view of the different actors in the tourism industry, measures of the quality of tourism service, and different measures of impact, such as the measure of the impact of tourism on the economy of countries. On the other hand, the keyword “sustainable-tourism,” which appears more frequently in the second period, shows the growing interest in sustainability issues in the management of tourism services. Something similar happens with the word “tourism-destination,” which is more frequent in the second period than in the first, reflecting the analysis of tourism destinations from more and more perspectives. Finally, the keyword “word-of-mouth” shows the growing importance in the analysis of tourism services of the opinions of tourists on online platforms for booking accommodation and tourism services, on social networks, discussion forums, and others. It is also referred to as e-WOM (electronic Word of mouth). It is a keyword also more frequent in the second period than in the first.

### Information Sources

In first place, we find “information-sources” or “tourist-information-system” among the keywords with the highest number of documents. Also noteworthy is the keyword “GIS” which refers to the high use of spatial data in tourism research or “big-data” which reflects the use of large datasets.

Reviewing the keywords in more depth, we find notable changes between the periods in the sources of information used, as we have already seen in the evolution map. For example, the keyword “online-reviews” and others with the same meaning “online-customer-reviews” or “online-travel-reviews” appear with a high frequency in the second period, while they do not appear in the first period. This keyword refers to the reviews made by tourists on online platforms for booking accommodation and tourist services (booking, Airbnb, tripadvisor), which are increasingly used as sources of information in data-driven tourism research.

**Fig. 12:**
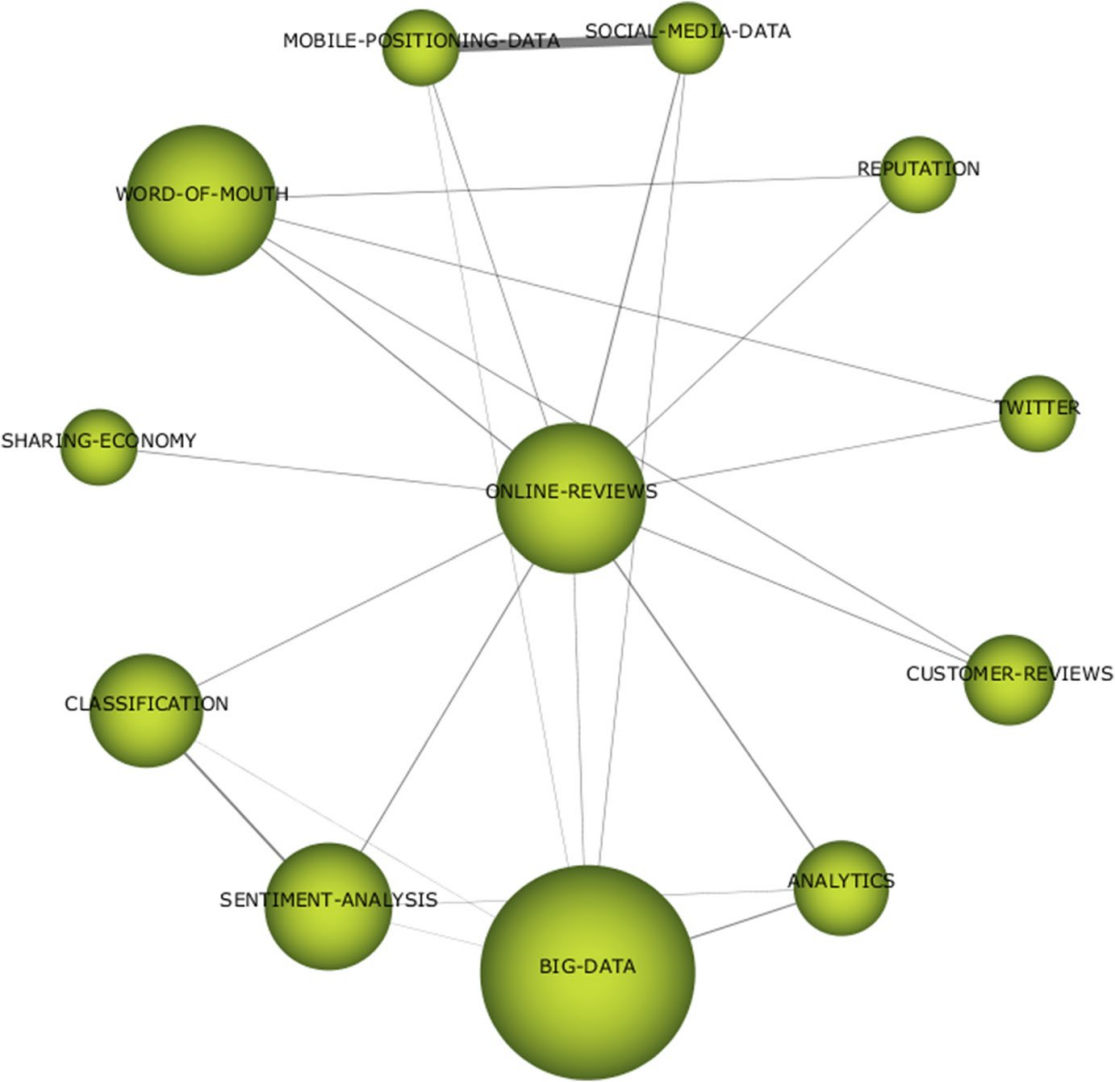
Keywords in theme “online-reviews”

It is interesting to take a closer look at the “online-reviews” cluster in the second period, already mentioned in “First Period (1982–2015)” above. In Fig. 12, we can see some of the sources where the data is generated (“twitter,” “customer-reviews,” “word-of-mouth), related information sources (“mobile-positioning-data,” “social-media-data”), analytical techniques (“analytics,” “big-data,” “sentiment-analysis,” “classification”), and topics to which research based on this data is applied (“reputation,” “sharing-economy”).

As indicated above, data of the type “online-reviews” are more generally classified in the UGC (user-generated content) data. Similarly, the keyword “user-generated-content” appears in the second period, but not in the first. Something similar happens with keywords referring to social networks or images, with a notably higher usage in the second period. Other sources of information that are also growing are data related to IoT (Internet of Things), mobile device data or trends in Internet search engines, although in the latter ones the difference between the first and second period, reviewing the keywords, is not so notable. Similarly, the keyword “big-data” is clearly a second-period keyword, reflecting the increased use of large datasets in tourism research in recent years.

As we have already mentioned, one of the keywords with the highest number of documents is “tourist-information-system.” In data-driven initiatives, integrated information systems are the key. In order to detect this type of systems that centralize information, we have searched for other keywords such as “data-warehouse” or “data-lake” and similar. Reviewing the documents associated with them, we found a relatively low development of this type of repositories in the tourism sector, where their use could be key.

With a similar objective, we searched for keywords referring to tourism statistics data, such as “tourism-statistics” or similar. We found a relatively low number of documents, reviewing the documents we found that tourism statistics data produced by different types of institutions has a relatively low use in data-driven tourism research.

We found something similar when we reviewed open data sources and searched for keywords such as “open-data.” We found a low number of documents that denotes a low use in research of this type of sources that have had a remarkable growth in the last decade.

## Data Analysis Techniques

**Fig. 13:**
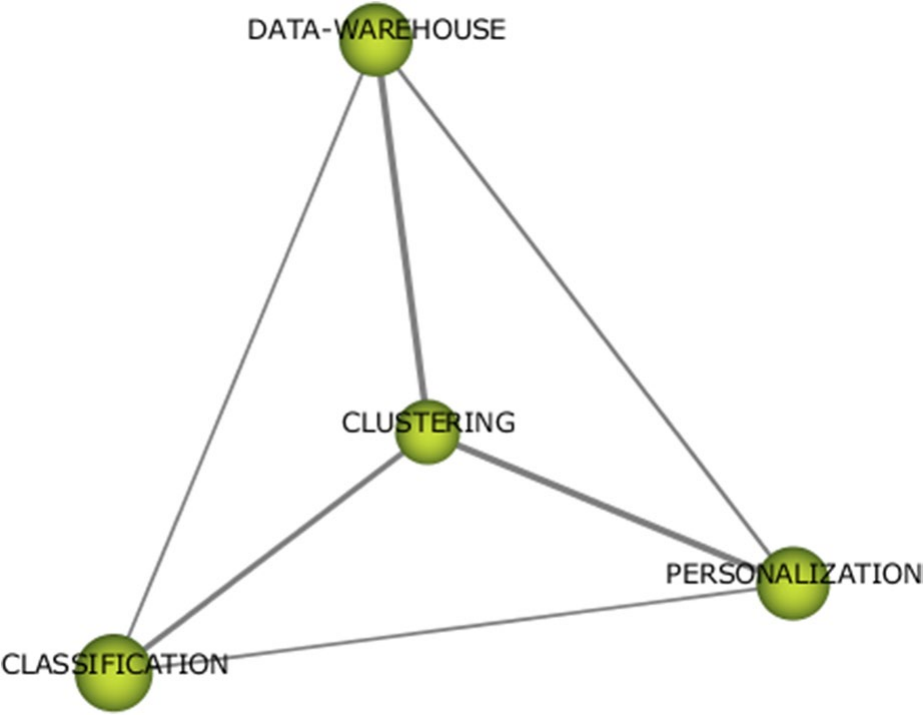
Keywords in theme “clustering”

**Fig. 14:**
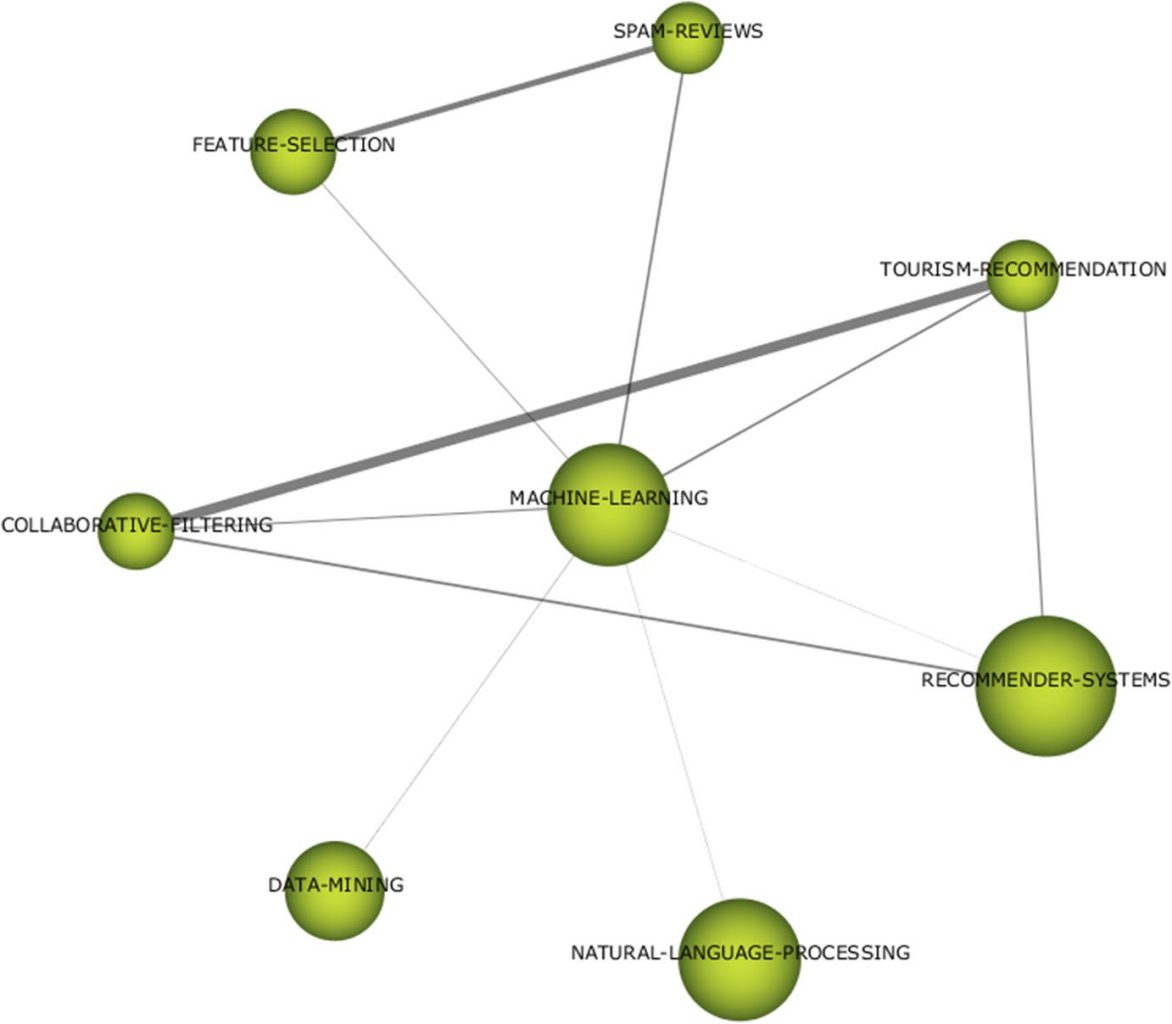
Keywords in theme “machine learning”

**Fig. 15:**
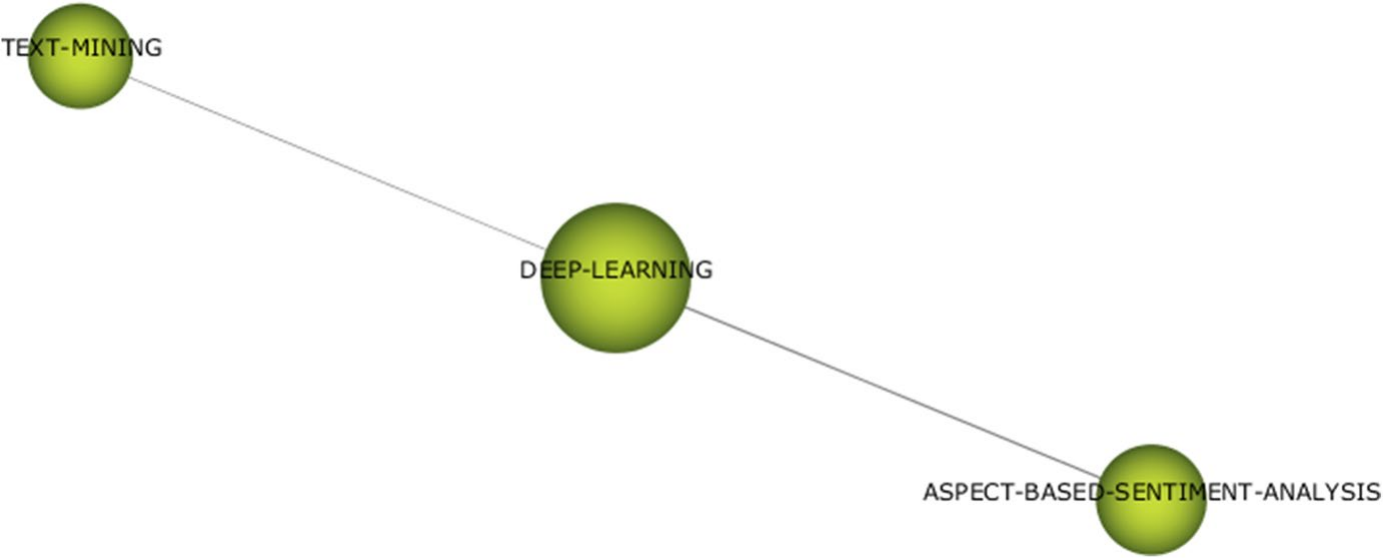
Keywords in theme “deep learning”

With regard to “data analysis techniques” thematic area, three themes have been detected: “clustering” in the first period and “machine learning” and “deep learning” in the second period (Figs. 13, 14, and 15). Since there are few themes and with very generic denominations, it is interesting to explore the keyword composition of each theme that we can obtain with SciMAT software.

Keywords related to more advanced or innovative analytical techniques, such as “machine learning,” “sentiment-analysis,” “text-mining,” “natural-language-processing,” “recommendation-systems,” “sentiment-analysis,” or “deep learning” appear much more frequently in the second period, reflecting the increasing use of advanced analytical techniques in tourism research, in accordance with what was seen in the evolution map.

### A Conceptual Model of Data Architecture and Processes of a Data-Driven Organization in the Tourism Sector

**Fig. 16:**
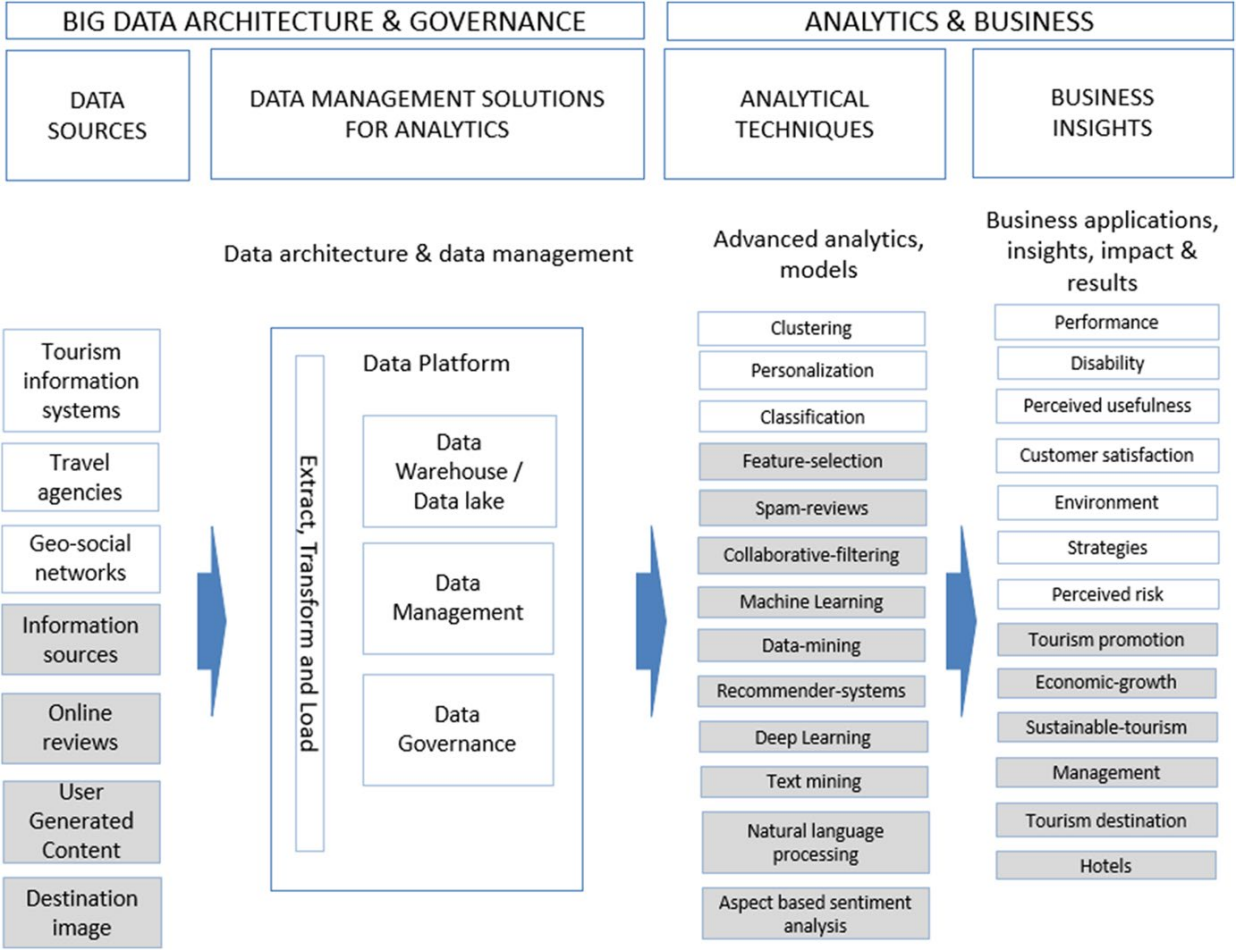
Conceptual model of data architecture and processes of a data-driven organization in the tourism sector

Once the major areas have been identified and analyzed, we are now going to translate these areas into the conceptual model of data and process architecture presented in Fig. 1 in the introduction section. This model showed the data and process architecture of a data-driven company, which, as established in the methodology, is our priority objective. We integrated each thematic area into the corresponding area in the model (data sources, analytical techniques, and business insights). By integrating the thematic areas detected in this model, we intend to show how this model is adapted to the tourism sector, as we can see in Fig. 16. It should be noted that, in the specific case of the themes of the “data analysis technique” thematic area, given that few and very generic themes had been detected, the keywords that make up each theme and that have been shown in the previous section have been incorporated into the model. The themes belonging to the second period are highlighted in gray to differentiate them from the first period.

This model identifies four main areas:

- Data sources: The most commonly used data sources in tourism research are identified. The evolution of information sources from one period to the next is shown. We obtain a wide range of different sources of information, among which we can highlight: tourism information systems (Puhretmair et al., 2002), geosocial networks (Yin et al., 2018), destination image (Chang & Chiang, 2022), online reviews (Kim & Han, 2022), or UGC (Li et al., 2018; Mukhopadhyay et al., 2022; Akbari et al., 2022). The information sources online reviews or UGC are currently the most widely used, which provide valuable information on tourist perception and behavior.
- Data management solutions: The model highlights the importance of having a data platform (Shi, 2020) that facilitates the storage and organization of data in a centralized and efficient way. There has been research in this area (Navarro et al., 2000; Abdulaziz et al., 2015; Ramos et al., 2017), but there is generally little research in this field and there is a lack of centralized data repositories in the tourism sector, especially data lake repositories
- Analytical techniques: The most commonly used analytical techniques in tourism research are identified. We obtain a wide range of analytical techniques, among which we can highlight: clustering (Feng et al., 2022), personalization (Gupta et al., 2022), collaborative-filtering (He, 2022), machine learning (Kayakus, 2022), recommender-systems (Julashokri et al., 2022), data-mining (Ma, 2022), text mining (Loureiro et al., 2022), natural language processing (Ray & Bala, 2021), or deep learning (law et al., 2019). some of the techniques identified like: text mining, sentimental analysis or natural language processing are applied to unstructured text-type information, which is the information typically collected in the sources like UGC or online reviews identified in the data sources area. It highlights the evolution towards machine learning or deep learning techniques.
- Business insights: The most important areas of business applications are identified. We obtain a wide range of business applications, among which we can highlight: customer satisfaction (Padma & Ahn, 2020), tourism promotion (Wu et al., 2022), sustainable tourism (Nilashi et al., 2019), or tourism destination (Rashad, 2022). The evolution of the areas from one period to the next is shown.

## Conclusions and Future Work

In this section, we show conclusions considering the theoretical and managerial implications and pointing out ideas for future research.

### Theoretical Implications

From an academic and research perspective, there are two main contributions. The primary objective, that is the proposed conceptual model of data architecture and processes, and the conclusions of the secondary objective, that is the bibliometric analysis

#### Conceptual Model of Data Architecture and Processes

This model identifies three areas of high interest to the scientific community.

- Data sources: The most commonly used data sources in tourism research are identified and should be taken into account in future research. Different types of information sources that can provide value are shown. Particular emphasis is placed on UGC type data, with great potential for the scientific community.
- Analytical techniques: The most commonly used analytical techniques in tourism research are identified and should be taken into account in future research. Techniques related to machine learning and deep learning are currently the most widely used in the scientific community.
- Business insights: The most important areas of scientific research are identified. These broad areas establish the main lines of research.

#### Conclusions of the Bibliometric Analysis

In this work, a bibliometric analysis of tourism research based on data sources has been carried out. WoS publications from 1982 to 2021 have been used. The following conclusions can be drawn:

Tourism research based on data sources is a field of research that has attracted the interest of the scientific community over the years, as evidenced by the remarkable growth of publications over the years.

Three major thematic areas are identified: “tourism research topics,” “information sources,” and “data analysis techniques.”

These three main areas are represented within a conceptual model of data architecture and processes of a data-driven organization in the tourism sector is obtained. This conceptual model shows a complete and useful view of data sources and analytical techniques used as well as business applications of a data-driven organization in tourism sector.

We have identified themes that have attracted the interest of the scientific community. If we focus on the first period: “performance,” “travel-agencies,” “tourism-information-systems,” and “customer satisfaction.” The themes identified in the second period and which could continue to be of interest in the coming years are: “determinants,” “tourism-destination,” “online-reviews,” and “management.”

Conceptual evolution maps have been obtained between the two periods in which we can see the evolution of the topics in the three identified thematic areas.

Authors, countries, and journals that have played a major role in the development of the research field have been identified. Pröll, Birgit, is the most productive author, USA isthe most productive country, and “*Tourism Management*” is the journal with the highest number of papers.

### Managerial Implications

The proposed conceptual model of data architecture and processes can be helpful for a company in the tourism sector that aims to become a data-driven company in its digital transformation process. This model identifies the most important sources of information to work on and the most commonly used analytical techniques. It also identifies the main business applications in tourism sector. These three areas serve as a guide for a data-driven company.

In the use of data sources, some recommendations that can come out of our study are the following:

The types of data sources used have evolved from the use of statistical or transactional data (bookings, travel) to the use of a broad spectrum of sources: GIS, IoT, images, social networks, search engine trends, opinions, and comments platforms. In this regard, it is worth noting the growing importance of UGC data (reviews, social networks, Google searches, etc.) and mobility data. This type of information makes it possible to analyze customer satisfaction or customer behavior. This type of analysis has been gaining importance and a transition has taken place that places the tourist at the center of the actions.

In the conceptual model, a data management solutions area is proposed. Regarding the use of this type of area, it has been detected a significant lack of centralized data repositories to collect and store data on a large scale, making use of appropriate data management techniques, ranging from traditional data warehouse structures to more flexible an agile data lake. The development of such repositories would centralize and simplify access to massive data sources and extend and democratize access to data sources to all agents in the tourism value chain.

Relatively, little use is detected in the studies of statistical information generated by institutions (Horobets, 2021) and open-data information that could have more potential and use. This type of information should be considered by data-driven companies as information external to the company, but with great potential.

### Ideas for Future Research

This study has some limitations. First, the results are supported by the WoS. Therefore, the limitations of the WoS should be considered. The search is based in core collection of WoS and did not include reports, dissertations, and other publication sources. This may be considered for future work to broaden the range of publication types in the study.

Likewise, the use of other bibliometric techniques that use other methods of relationships among publications or other performance indicators can complement the obtained results.

As future work, we propose going a step further in the application of the conceptual model of data architecture and processes obtained in this article. This conceptual model provides a general framework for companies in the tourism sector that want to be data-driven and more market-oriented. A second step in this model can be taken; based on the characteristics of each type of the data sources of the tourism sector, we can customize each component of the model as much as possible. That is, we can find the most appropriate data management solution for each type of data source and look for the most appropriate analytical techniques for each type of source and that generate the greatest impact on business insights. Likewise, it would be interesting to identify which type of insights have a greater direct benefit in the management of the tourism sector. These steps to customize the conceptual model could be applied to other sectors of activity (banking, insurance, etc.).
